# Supplementary material for: Genome-wide association study of resistance to Mycobacterium tuberculosis infection identifies a locus at 10q26.2 in three distinct populations
Source: PLoS Genet. 2021 Mar 4;17(3):e1009392. doi: 10.1371/journal.pgen.1009392 (PMC7963100; doi:10.1371/journal.pgen.1009392)
Supplement: S1 Text — Immune assays in Vietnam, France and South Africa. (PDF) [file pgen.1009392.s001.pdf]

Supplemental Methods

Immune assays in Vietnam

For tuberculin skin test (TST), 5 units (TU) of purified protein derivative (PPD) solution per 0.1 mL test dose (Tubertest, Sanofi Pasteur, Lyon, France) were injected intradermally into the forearm of the household contacts (HHCs) using the Mantoux method. The diameter of the induration was measured by trained personal 48-72 hours after the injection. QuantiFERON-TB Gold In-Tube test (QFT-GIT) assay was performed on blood samples collected before the TST was carried out, in accordance with the kit manufacturer's instructions. Briefly, peripheral blood was drawn directly into each of three blood collection tubes under vacuum: the nil tube or negative control, the mitogen tube containing phytohaemagglutinin (PHA) as a positive control and the TB antigen tube containing specific antigens for *M. tuberculosis* (early secretory antigenic target 6, ESAT6, culture filtrate protein 10 and TB 7.7 antigen). The contents of each tube were mixed and the tubes were incubated at 37°C for 16 to 24 hours within 8 hours of collection. Tubes were centrifuged at 2500 g for 15 minutes at room temperature, plasma was collected and stored at -20°C. The concentration of interferon-gamma (IFN-γ) in each sample was determined with the QFT-GIT ELISA (enzyme-linked immunosorbent assay) kit. Results were categorized by the manufacturer's software as positive, negative or indeterminate.

Immune assays in France

TSTs were performed on the volar surface of the forearm by intradermal injection of 0.1 mL (5 TU) of purified protein derivative tuberculin obtained from a human *M. tuberculosis* strain (Tubertest, Sanofi Pasteur, Lyon, France). The skin induration was read within 72 hours. IGRAs were performed once either at V1 or V2. Blood was drawn from each individual and peripheral blood mononuclear cells (PBMCs) were separated. They were activated with one of the following stimulations: ESAT-6 (rdESAT6, Statens-Serum-Institute, Denmark; 2.5µg/mL),

PHA (6.25 µg/mL) for positive control and medium alone for negative control. After 4 days of stimulation, supernatants were assayed for IFN-γ by ELISA according to the manufacturer's recommendations (Pelikin Compact; CLB). The final results were standardized per million PBMC's, in the unit pg/mL/10^6 PBMC's.

Immune assays in South Africa

TSTs were performed by specially trained health care providers using the Mantoux method with PPD solution (2 T.U Tuberculin PPD RT 23, Statens Serum Institut, Copenhagen, Denmark) and skin induration was read 48-72 hours later. Whole-blood specimens were stimulated by ESAT-6 (rdESAT6, Statens-Serum-Institute, Denmark; 20µg/mL) and PHA (5 µg/mL) for positive control. Cultures incubated without stimulation in serum-free medium provided the negative control. IFN-γ levels were measured on day 3 after stimulation (limit of detection at 31 pg/mL).
