# Supplementary material for: Genome-wide association study of resistance to Mycobacterium tuberculosis infection identifies a locus at 10q26.2 in three distinct populations
Source: PLoS Genet. 2021 Mar 4;17(3):e1009392. doi: 10.1371/journal.pgen.1009392 (PMC7963100; doi:10.1371/journal.pgen.1009392)
Supplement: S1 Fig — Plot of the expected distribution of association test statistics (x axis) for 5,591,951 variants compared to the observed values (y axis). Red line is the null hypothesis of no association (y = x). (PDF) [file pgen.1009392.s002.pdf]

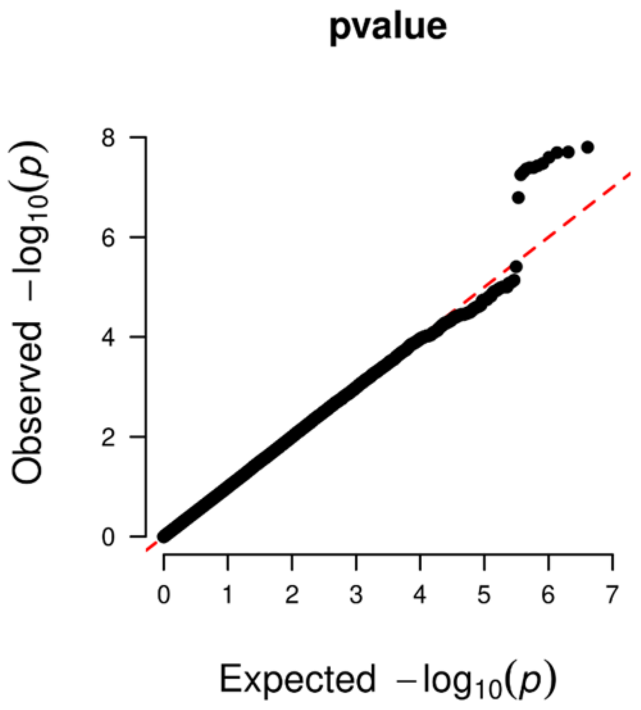

S1 Figure. Quantile-quantile plot of GWAS resistance to tuberculosis infection in Vietnam. Plot of the expected distribution of association test statistics (x axis) for 5,591,951 variants compared to the observed values (y axis). Red line is the null hypothesis of no association (y = x).
