# Supplementary material for: Genome-wide association study of resistance to Mycobacterium tuberculosis infection identifies a locus at 10q26.2 in three distinct populations
Source: PLoS Genet. 2021 Mar 4;17(3):e1009392. doi: 10.1371/journal.pgen.1009392 (PMC7963100; doi:10.1371/journal.pgen.1009392)
Supplement: S3 Fig — Pairwise LD coefficients r2 are shown in each cell between 18 variants with P < 5.0 × 10^–6^. Top genotyped variant rs17155120 is second from left. (PDF) [file pgen.1009392.s004.pdf]

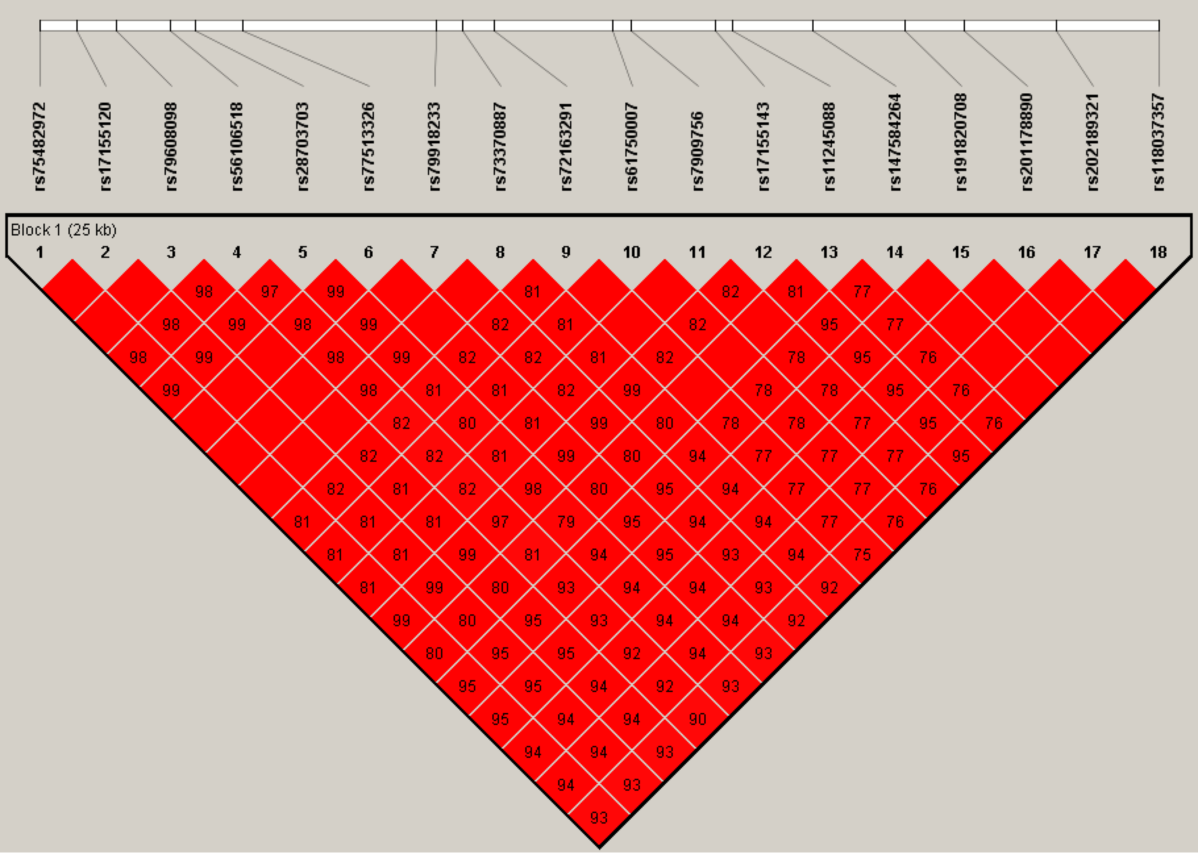

S3 Figure. Haploview LD graph of the top associations on chromosome 10q26.2 in Vietnam. Pairwise LD coefficients r^2 are shown in each cell between 18 variants with $P < 5.0 \times 10^{-6}$. Top genotyped variant rs17155120 is second from left.
