# Supplementary material for: Genome-wide association study of resistance to Mycobacterium tuberculosis infection identifies a locus at 10q26.2 in three distinct populations
Source: PLoS Genet. 2021 Mar 4;17(3):e1009392. doi: 10.1371/journal.pgen.1009392 (PMC7963100; doi:10.1371/journal.pgen.1009392)
Supplement: S4 Fig — (PDF) [file pgen.1009392.s005.pdf]

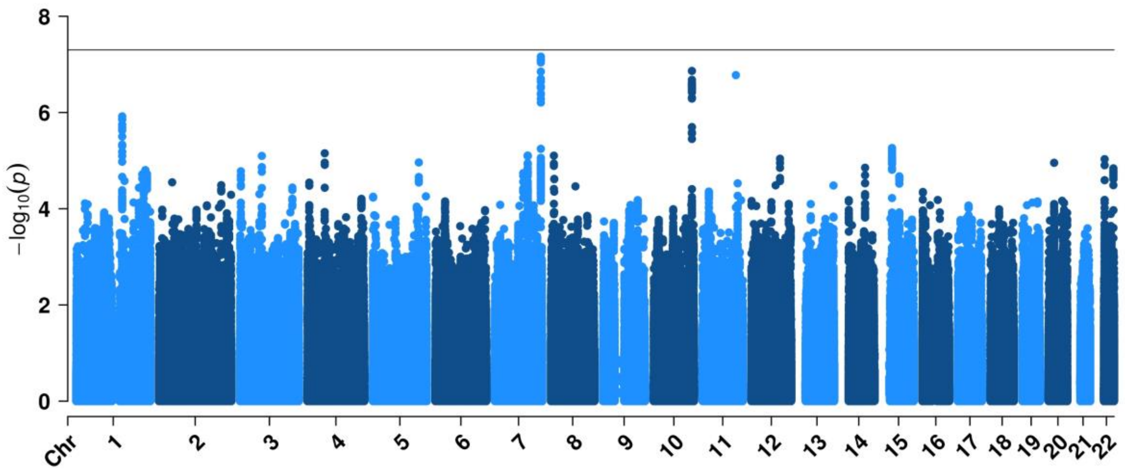

S4 Figure. Manhattan plot for the GWAS of resistance to *M. tuberculosis* infection in Vietnam (185 uninfected vs 201 infected participants).
