# Supplementary material for: Genome-wide association study of resistance to Mycobacterium tuberculosis infection identifies a locus at 10q26.2 in three distinct populations
Source: PLoS Genet. 2021 Mar 4;17(3):e1009392. doi: 10.1371/journal.pgen.1009392 (PMC7963100; doi:10.1371/journal.pgen.1009392)
Supplement: S5 Fig — Pairwise LD coefficients r2 are shown in each cell A) between the 17 variants of the locus in the French cohort, and B) between 12 out of the 18 variants of the locus in the South African cohort. Top genotyped variant rs17155120 is second from left in each figure. (PDF) [file pgen.1009392.s006.pdf]

A) France **B) South Africa**

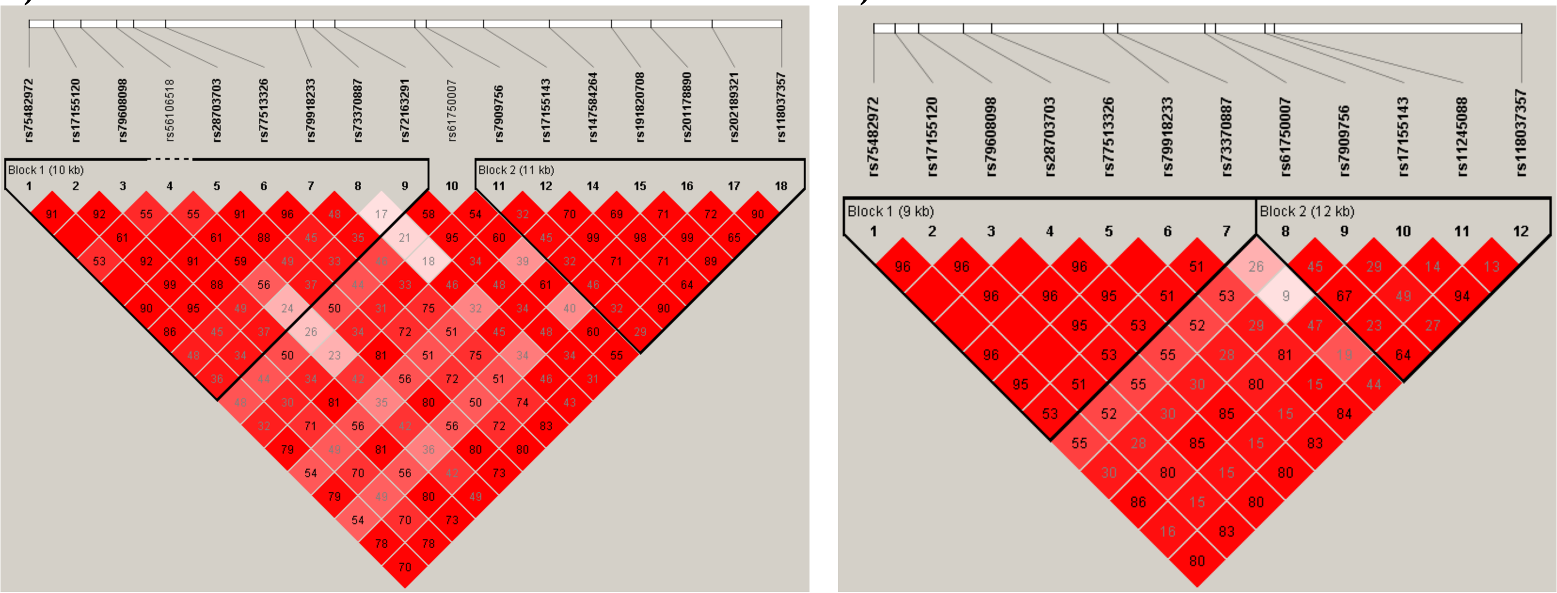

S5 Figure. Haploview LD graphs of the locus on chromosome 10q26.2 in replication cohorts. Pairwise LD coefficients r^2 are shown in each cell **A)** between the 17 variants of the locus in the French cohort, and **B)** between 12 out of the 18 variants of the locus in the South African cohort. Top genotyped variant rs17155120 is second from left in each figure.
