# Supplementary material for: Genome-wide association study of resistance to Mycobacterium tuberculosis infection identifies a locus at 10q26.2 in three distinct populations
Source: PLoS Genet. 2021 Mar 4;17(3):e1009392. doi: 10.1371/journal.pgen.1009392 (PMC7963100; doi:10.1371/journal.pgen.1009392)
Supplement: S6 Fig — The T allele (in blue) is protective against M. tuberculosis infection. Map generated from the Geography of Genetic Variants Browser (http://www.popgen.uchicago.edu/ggv). (PDF) [file pgen.1009392.s007.pdf]

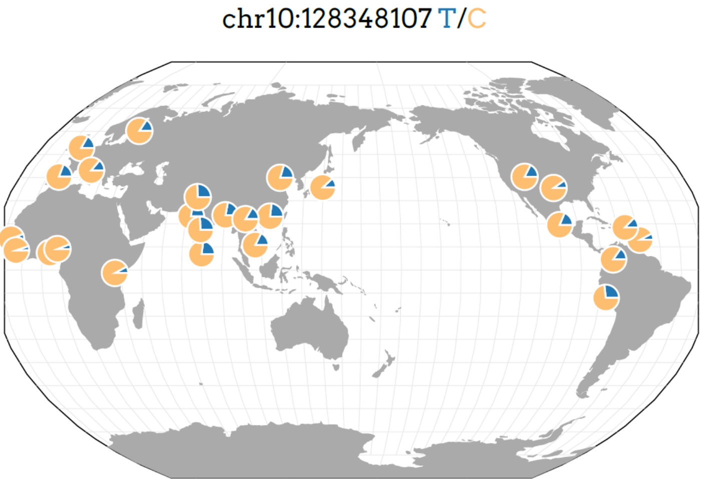

S6 Figure. Geographic distribution of the variant rs17155120 in 1000G phase 3 populations. The T allele (in blue) is protective against *M. tuberculosis* infection. Map generated from the Geography of Genetic Variants Browser (http://www.popgen.uchicago.edu/ggv).
