# Supplementary material for: Genome-wide association study of resistance to Mycobacterium tuberculosis infection identifies a locus at 10q26.2 in three distinct populations
Source: PLoS Genet. 2021 Mar 4;17(3):e1009392. doi: 10.1371/journal.pgen.1009392 (PMC7963100; doi:10.1371/journal.pgen.1009392)
Supplement: S7 Fig — Pairwise linkage disequilibrium (r2) in A) Vietnamese, B) French and C) South African study cohorts (left panels) between 60 variants in a 30 kb window around rs17155120 (arrow) as compared to 1000G populations (right panels). (PDF) [file pgen.1009392.s008.pdf]

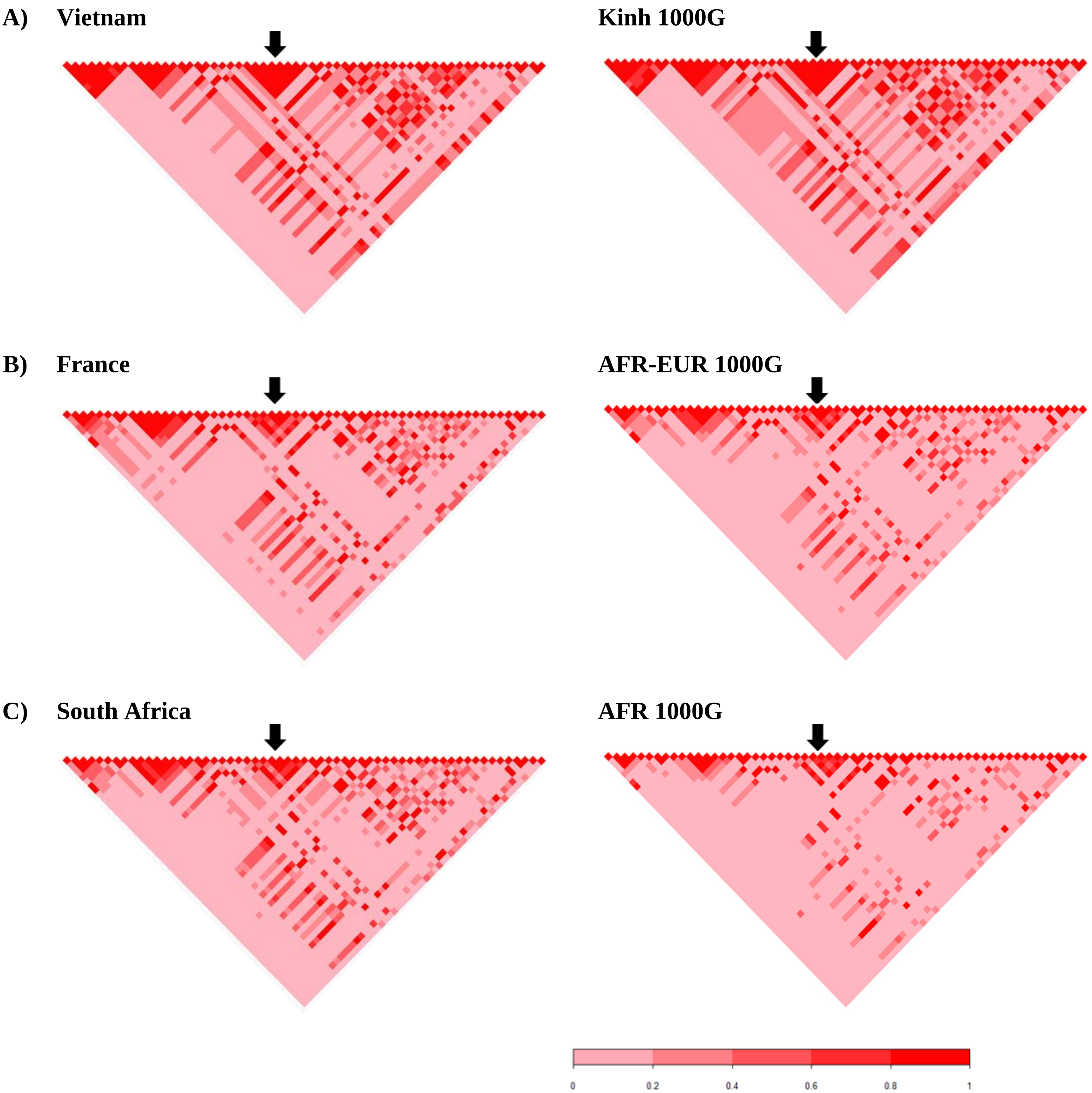

S7 Figure. Pairwise linkage disequilibrium (r^2) in A) Vietnamese, B) French and C) South African study cohorts (left panels) between 60 variants in a 30 kb window around rs17155120 (arrow) as compared to 1000G populations (right panels).
