# Supplementary material for: Genome-wide association study of resistance to Mycobacterium tuberculosis infection identifies a locus at 10q26.2 in three distinct populations
Source: PLoS Genet. 2021 Mar 4;17(3):e1009392. doi: 10.1371/journal.pgen.1009392 (PMC7963100; doi:10.1371/journal.pgen.1009392)
Supplement: S8 Fig — (PDF) [file pgen.1009392.s009.pdf]

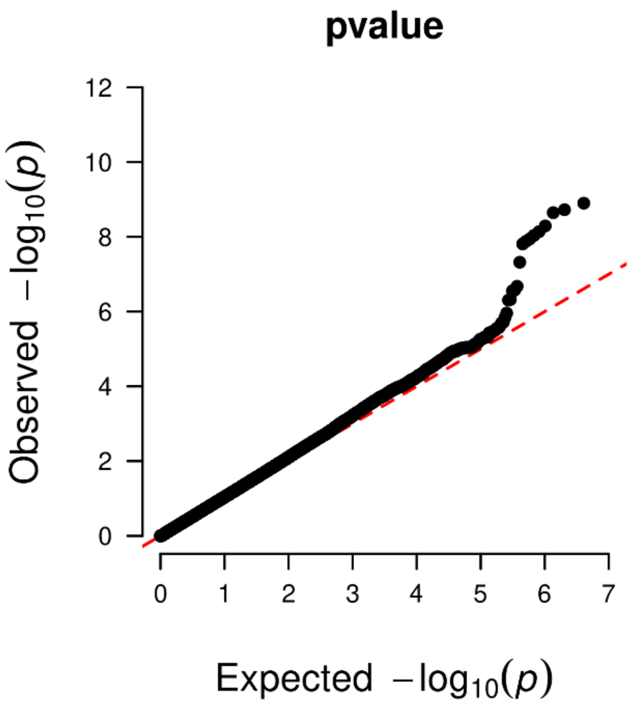

S8 Figure. Quantile-quantile plot of GWAS of resistance to *M. tuberculosis* infection in the 3 cohorts from Vietnam, France and South Africa (333 uninfected vs 616 infected subjects) (λ = 1.06).
