# Supplementary figures and images for: Genome-wide association study of resistance to Mycobacterium tuberculosis infection identifies a locus at 10q26.2 in three distinct populations
Source: PLoS Genet. 2021 Mar 4;17(3):e1009392. doi: 10.1371/journal.pgen.1009392 (PMC7963100; doi:10.1371/journal.pgen.1009392)

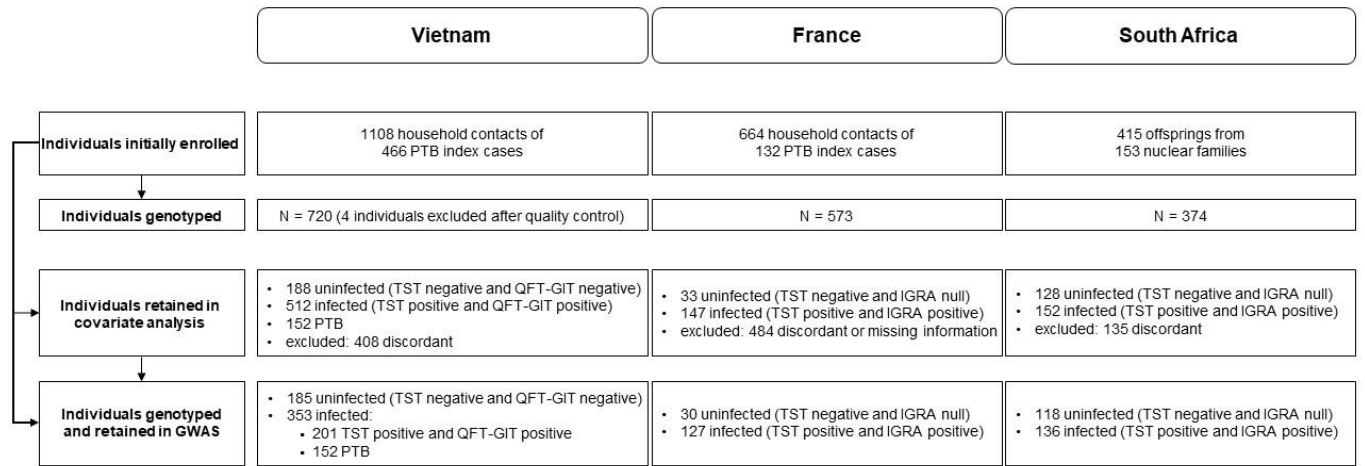

**S9 Figure. Flowchart of the study.**

Supplement: S9 Fig — (PDF) [file pgen.1009392.s010.pdf]
