# Supplementary material for: Genome-wide association study of resistance to Mycobacterium tuberculosis infection identifies a locus at 10q26.2 in three distinct populations
Source: PLoS Genet. 2021 Mar 4;17(3):e1009392. doi: 10.1371/journal.pgen.1009392 (PMC7963100; doi:10.1371/journal.pgen.1009392)
Supplement: S10 Fig — Plot of the first and second principal components of the 720 individuals from the Vietnamese cohort after projection A) on the 1000 Genomes phase 3 population, B) on the East Asian 1000 Genomes Phase 3 populations only. (PDF) [file pgen.1009392.s011.pdf]

A)

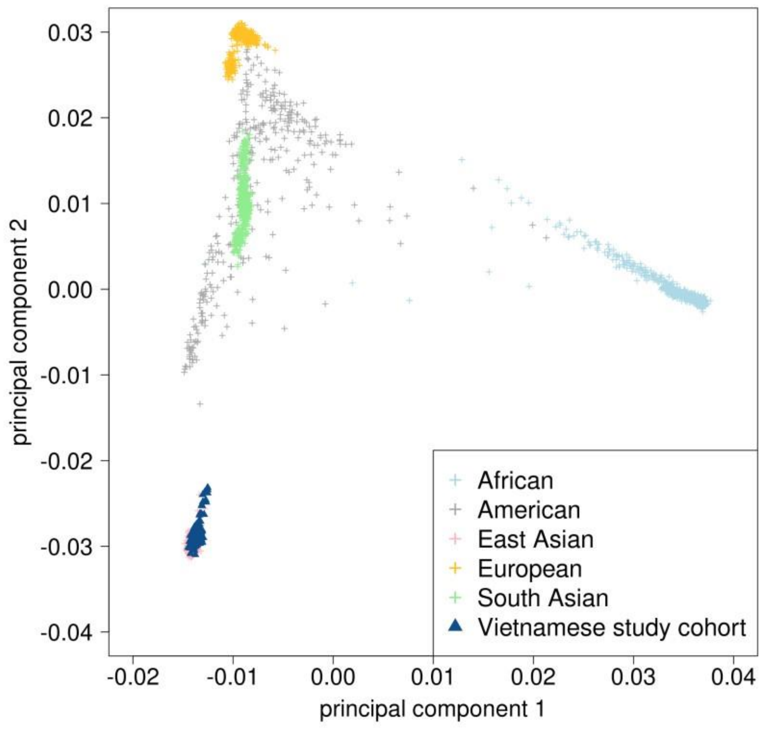

B)

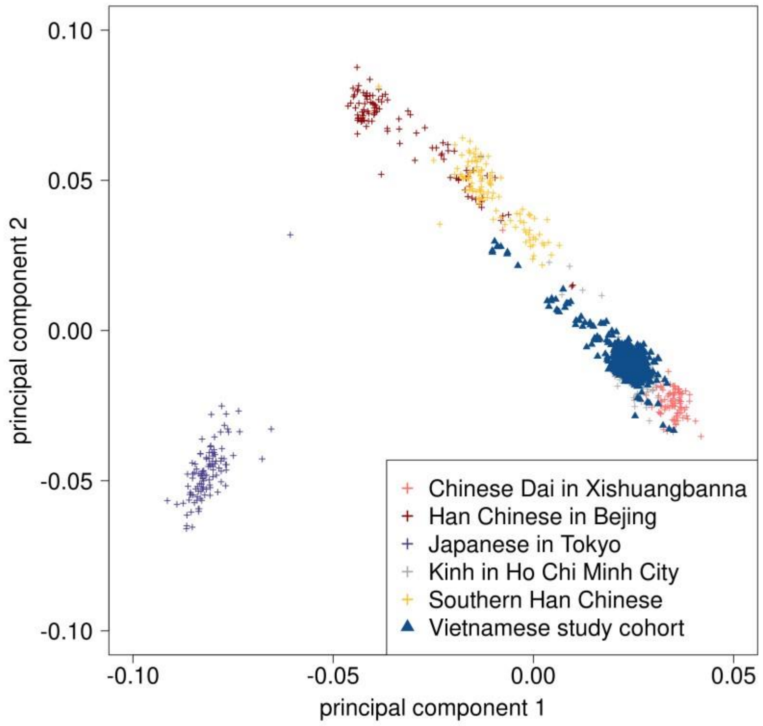

S10 Figure. Principal component analysis of the Vietnamese study cohort. Plot of the first and second principal components of the 720 individuals from the Vietnamese cohort after projection **A)** on the 1000 Genomes phase 3 population, **B)** on the East Asian 1000 Genomes Phase 3 populations only.
