# Supplementary material for: Genome-wide association study of resistance to Mycobacterium tuberculosis infection identifies a locus at 10q26.2 in three distinct populations
Source: PLoS Genet. 2021 Mar 4;17(3):e1009392. doi: 10.1371/journal.pgen.1009392 (PMC7963100; doi:10.1371/journal.pgen.1009392)
Supplement: S11 Fig — Plot of the first and second principal components of A) 573 individuals from the French cohort and B) the 157 subjects subsequently analyzed in the GWAS, after projection on the 1000 Genomes phase 3 populations. (PDF) [file pgen.1009392.s012.pdf]

A)

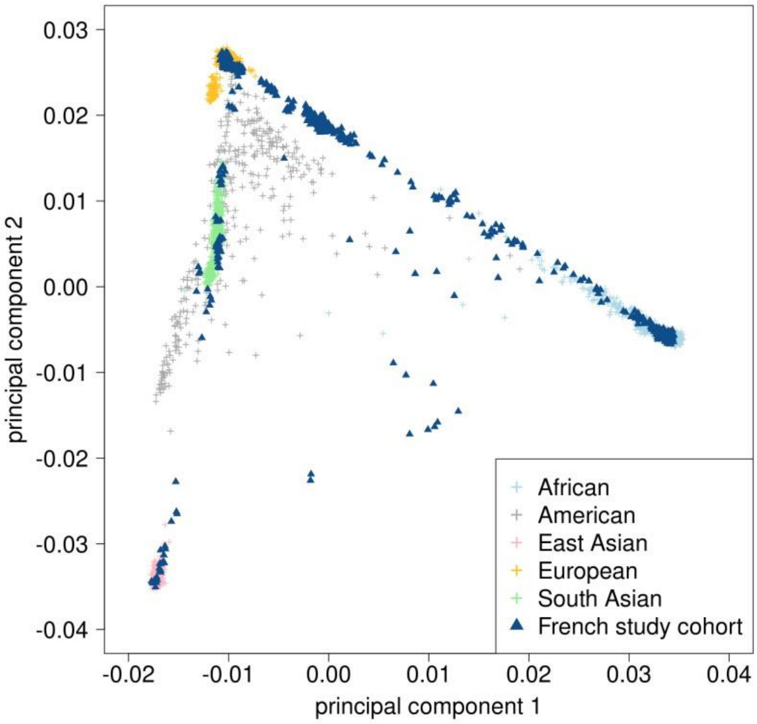

B)

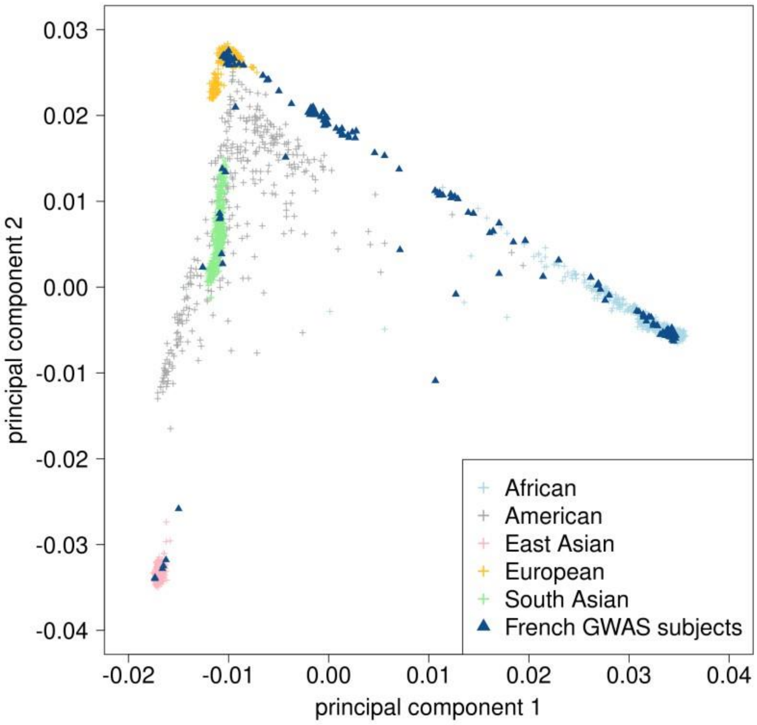

S11 Figure. Principal component analysis of the French cohort. Plot of the first and second principal components of **A)** 573 individuals from the French cohort and **B)** the 157 subjects subsequently analyzed in the GWAS, after projection on the 1000 Genomes phase 3 populations.
