# Supplementary material for: Genome-wide association study of resistance to Mycobacterium tuberculosis infection identifies a locus at 10q26.2 in three distinct populations
Source: PLoS Genet. 2021 Mar 4;17(3):e1009392. doi: 10.1371/journal.pgen.1009392 (PMC7963100; doi:10.1371/journal.pgen.1009392)
Supplement: S12 Fig — Plot of the first and second principal components of 374 individuals from the South African cohort after projection on the 1000 Genomes phase 3 populations. (PDF) [file pgen.1009392.s013.pdf]

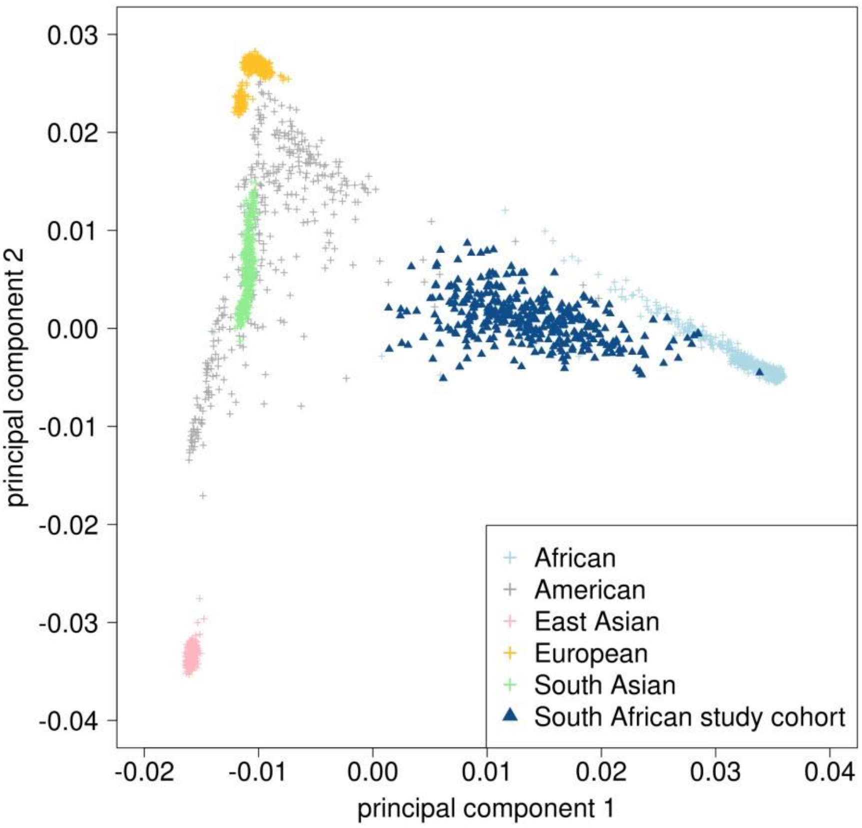

S12 Figure. Principal component analysis of the South African cohort. Plot of the first and second principal components of 374 individuals from the South African cohort after projection on the 1000 Genomes phase 3 populations.
