# Supplementary material for: Genome-wide association study of resistance to Mycobacterium tuberculosis infection identifies a locus at 10q26.2 in three distinct populations
Source: PLoS Genet. 2021 Mar 4;17(3):e1009392. doi: 10.1371/journal.pgen.1009392 (PMC7963100; doi:10.1371/journal.pgen.1009392)
Supplement: S13 Fig — The dashed line represents a 5 mm cut-off for TST. Uninfected subjects (in yellow) were negative for TST and QuantiFERON-TB Gold In-Tube (QFT-GIT) and infected subjects (in blue) positive for both. (PDF) [file pgen.1009392.s014.pdf]

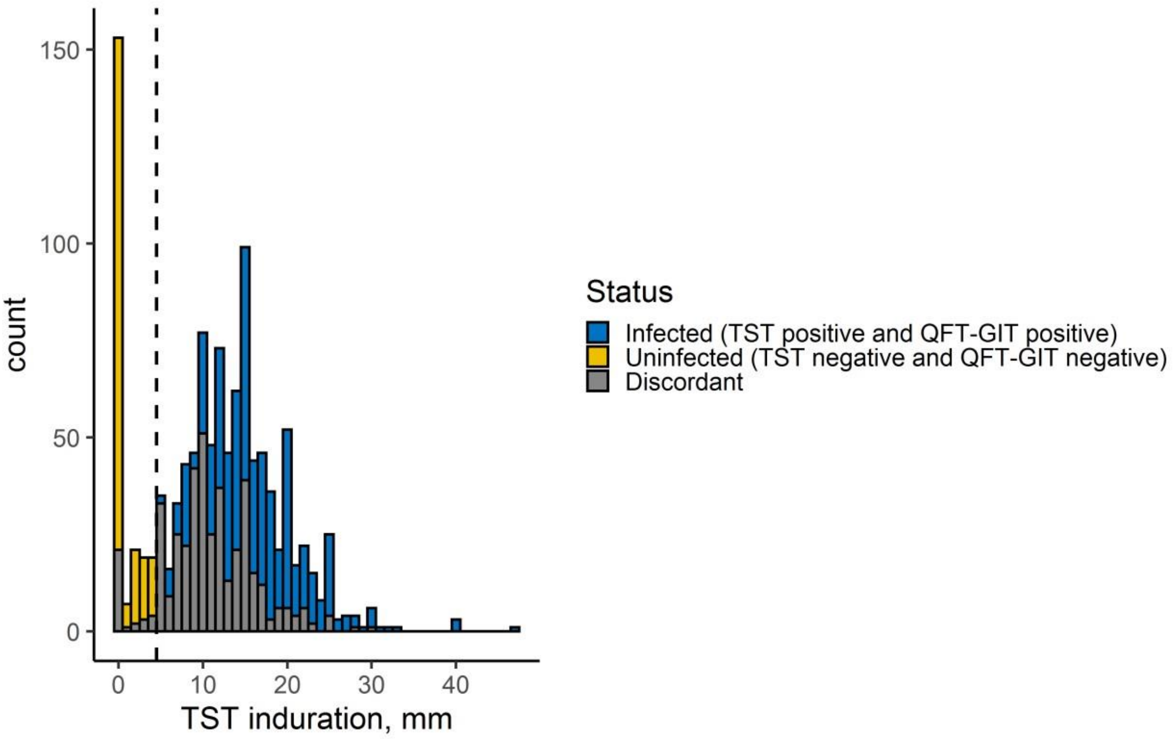

S13 Figure. Stacked histogram of the tuberculin skin test (TST) distribution, stratified by our infection definition among 1108 household contacts in Vietnam. The dashed line represents a 5 mm cut-off for TST. Uninfected subjects (in yellow) were negative for TST and QuantiFERON-TB Gold In-Tube (QFT-GIT) and infected subjects (in blue) positive for both.
