# Supplementary material for: Genome-wide association study of resistance to Mycobacterium tuberculosis infection identifies a locus at 10q26.2 in three distinct populations
Source: PLoS Genet. 2021 Mar 4;17(3):e1009392. doi: 10.1371/journal.pgen.1009392 (PMC7963100; doi:10.1371/journal.pgen.1009392)
Supplement: S14 Fig — A) Distribution of the tuberculin skin test (TST) induration among 516 HHCs (mean induration when 2 screening visits were done). The dashed line represents a 5 mm cut-off. TST was considered negative when the skin induration was i) < 5 mm at both V1 and V2, ii) < 5 mm at V1, when only one visit was done. TST was considered positive when the skin induration was i) ≥ 5 mm at both V1 and V2, ii) < 5 mm at V1 and ≥ 10 mm at V2. B) Construction of a ROC curve based on the defined TST status to determine the optimal interferon-γ release assay (IGRA) cut-off (175 pg/mL). C) Stacked histogram of the TST distribution stratified by our infection definition. Uninfected subjects (in yellow) presented a negative TST and a null production of IFN-γ. Infected subjects (in blue) presented a positive TST and a positive IGRA (IFN-γ production > 175 pg/mL). (PDF) [file pgen.1009392.s015.pdf]

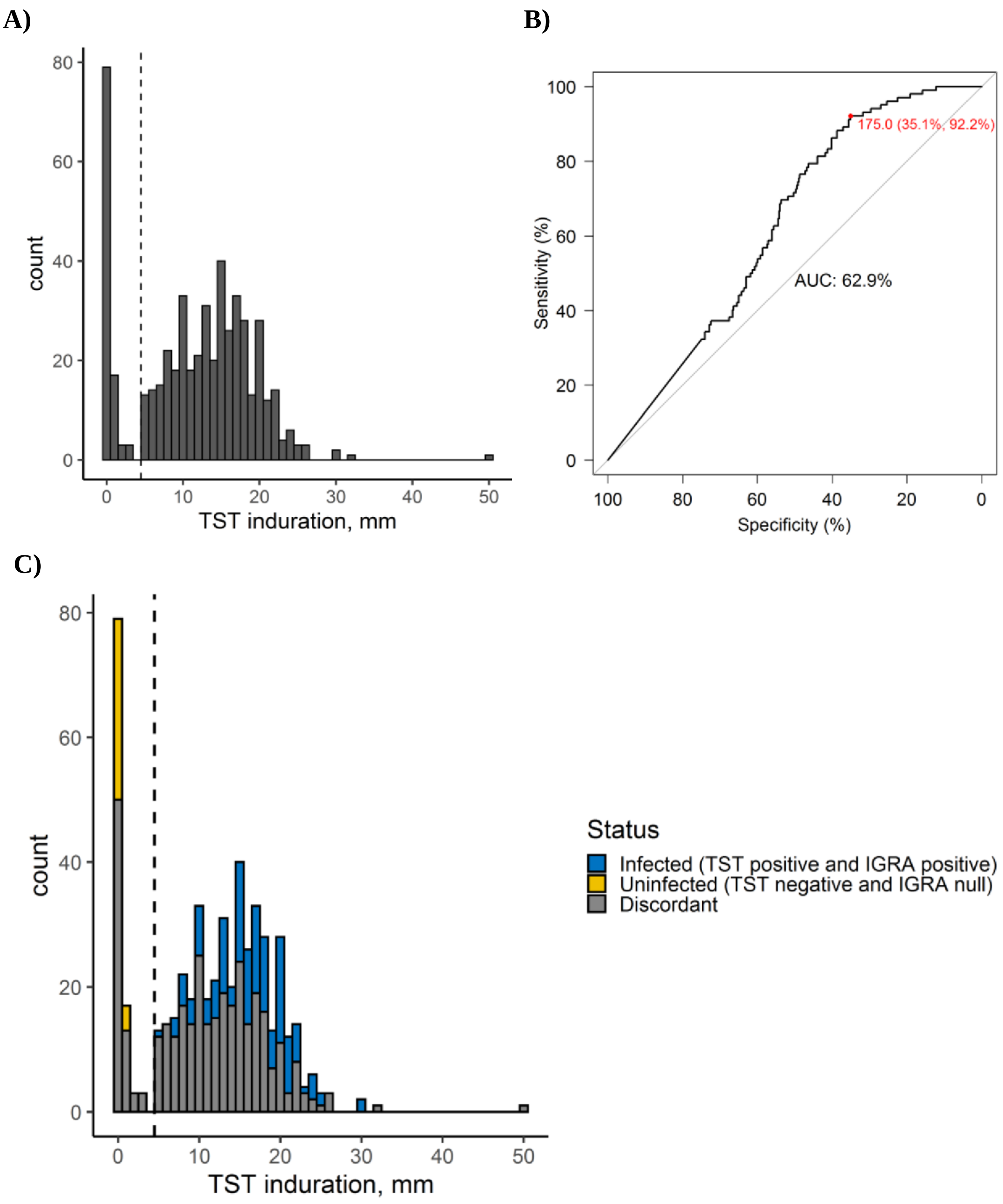

S14 Figure. Definition of the GWAS phenotype in the household contacts (HHCs) study in France. A) Distribution of the tuberculin skin test (TST) induration among 516 HHCs (mean induration when 2 screening visits were done). The dashed line represents a 5 mm cut-off. TST was considered negative when the skin induration was i) < 5 mm at both V1 and V2, ii) < 5 mm at V1, when only one visit was done. TST was considered positive when the skin induration was i) ≥ 5 mm at both V1 and V2, ii) < 5 mm at V1 and ≥ 10 mm at V2. **B)** Construction of a ROC curve based on the defined TST status to determine the optimal interferon-γ release assay (IGRA) cut-off (175 pg/mL). **C)** Stacked histogram of the TST distribution stratified by our infection definition. Uninfected subjects (in yellow) presented a negative TST and a null production of IFN-γ. Infected subjects (in blue) presented a positive TST and a positive IGRA (IFN-γ production > 175 pg/mL).
