# Supplementary material for: Genome-wide association study of resistance to Mycobacterium tuberculosis infection identifies a locus at 10q26.2 in three distinct populations
Source: PLoS Genet. 2021 Mar 4;17(3):e1009392. doi: 10.1371/journal.pgen.1009392 (PMC7963100; doi:10.1371/journal.pgen.1009392)
Supplement: S1 Table — (PDF) [file pgen.1009392.s017.pdf]

S1 Table. Info score of the associated variants on 10q26.2.

Variant	chr:pos	Vietnam	France	South Africa
rs11245088	10:128362986	0.91	-	0.92
rs72163291	10:128357575	0.92	0.92	-
rs17155143	10 :128362587	0.93	0.94	0.93
rs7909756	10:128360681	0.92	0.92	0.92
rs28703703	10:128350796	0.99	0.98	0.99
rs56106518	10:128350239	0.86	0.83	-
rs75482972	10:128347258	0.99	0.98	0.96
rs17155120	10:128348107	genotyped	genotyped	genotyped
rs73370887	10:128356858	0.95	0.96	0.93
rs79608098	10:128349019	0.99	0.99	0.99
rs61750007	10:128360275	0.94	0.95	0.94
rs77513326	10:128351876	0.99	0.99	genotyped
rs79918233	10:128356265	0.97	0.98	0.97
rs147584264	10:128364818	0.92	0.94	-
rs191820708	10:128366904	0.91	0.93	-
rs201178890	10:128368249	0.92	0.94	-
rs202189321	10:128370320	0.90	0.93	-
rs118037357	10:128372646	0.93	0.97	0.95
