# Supplementary material for: Genome-wide association study of resistance to Mycobacterium tuberculosis infection identifies a locus at 10q26.2 in three distinct populations
Source: PLoS Genet. 2021 Mar 4;17(3):e1009392. doi: 10.1371/journal.pgen.1009392 (PMC7963100; doi:10.1371/journal.pgen.1009392)
Supplement: S2 Table — (PDF) [file pgen.1009392.s018.pdf]

S2 Table. Variants in the locus on chromosome 10q26.2 associated with resistance to *M. tuberculosis* infection in Vietnam (185 uninfected and 201 double positive infected subjects).

Variant	EA	EAF	OR (95%CI)	*P* value
rs56106518	C	0.24	0.39 (0.30-0.47)	1.37×10^{-7}
rs72163291	ins	0.26	0.41 (0.33-0.49)	2.09×10^{-7}
rs28703703	G	0.21	0.40 (0.32-0.49)	2.40×10^{-7}
rs17155120	T	0.21	0.40 (0.32-0.49)	2.55×10^{-7}
rs11245088	C	0.26	0.41 (0.40-0.42)	2.72×10^{-7}
rs7909756	G	0.26	0.41 (0.33-0.49)	2.73×10^{-7}
rs75482972	A	0.21	0.41 (0.32-0.49)	2.82×10^{-7}
rs17155143	A	0.21	0.39 (0.30-0.47)	3.02×10^{-7}
rs79608098	T	0.21	0.41 (0.32-0.49)	3.28×10^{-7}
rs77513326	A	0.21	0.40 (0.32-0.49)	3.56×10^{-7}
rs73370887	A	0.21	0.39 (0.31-0.48)	3.82×10^{-7}
rs61750007	C	0.25	0.42 (0.34-0.50)	4.91×10^{-7}
rs79918233	A	0.21	0.40 (0.31-0.49)	5.13×10^{-7}
rs147584264	C	0.21	0.41 (0.32-0.50)	2.01×10^{-6}
rs191820708	A	0.21	0.41 (0.32-0.50)	2.59×10^{-6}
rs201178890	T	0.21	0.41 (0.32-0.50)	2.71×10^{-6}
rs202189321	T	0.21	0.41 (0.32-0.50)	2.72×10^{-6}
rs118037357	A	0.20	0.42 (0.32-0.51)	3.54×10^{-6}

CI, confidence intervals; EA, effect allele; EAF, effect allele frequency; OR, odds ratio; ins, insertion
