# Supplementary material for: Genome-wide association study of resistance to Mycobacterium tuberculosis infection identifies a locus at 10q26.2 in three distinct populations
Source: PLoS Genet. 2021 Mar 4;17(3):e1009392. doi: 10.1371/journal.pgen.1009392 (PMC7963100; doi:10.1371/journal.pgen.1009392)
Supplement: S3 Table — (PDF) [file pgen.1009392.s019.pdf]

S3 Table. Frequency of rs17155120 minor allele T in 1000 Genomes phase 3 populations[1].

1000 Genomes population	rs17155120 T frequency
ALL	0.16
African	0.07
Yoruba in Ibadan, Nigeria	0.07
Luhya in Webuye, Kenya	0.08
Gambian in Western Divisions in the Gambia	0.06
Mende in Sierra Leone	0.05
Esan in Nigeria	0.06
Americans of African Ancestry in SW USA	0.11
African Caribbeans in Barbados	0.08
American	0.18
Mexican Ancestry from Los Angeles USA	0.19
Puerto Ricans from Puerto Rico	0.12
Colombians from Medellin, Colombia	0.15
Peruvians from Lima, Peru	0.27
East Asian	0.18
Han Chinese in Beijing, China	0.20
Japanese in Tokyo, Japan	0.12
Southern Han Chinese	0.22
Chinese Dai in Xishuangbanna, China	0.16
Kinh in Ho Chi Minh City, Vietnam	0.18
European	0.16
Utah Residents with Northern and Western European Ancestry	0.18
Toscani in Italia	0.14
Finnish in Finland	0.16
British in England and Scotland	0.16
Iberian Population in Spain	0.19
South Asian	0.24
Gujarati Indian from Houston, Texas	0.22
Punjabi from Lahore, Pakistan	0.26
Bengali from Bangladesh	0.22
Sri Lankan Tamil from the UK	0.23
Indian Telugu from the UK	0.25

Reference

1. Machiela, M.J., and Chanock, S.J. (2015). LDlink: a web-based application for exploring population-specific haplotype structure and linking correlated alleles of possible functional variants. Bioinformatics *31*, 3555–3557
