# Supplementary material for: Genome-wide association study of resistance to Mycobacterium tuberculosis infection identifies a locus at 10q26.2 in three distinct populations
Source: PLoS Genet. 2021 Mar 4;17(3):e1009392. doi: 10.1371/journal.pgen.1009392 (PMC7963100; doi:10.1371/journal.pgen.1009392)
Supplement: S4 Table — (PDF) [file pgen.1009392.s020.pdf]

S4 Table. Variants associated with resistance to *M. tuberculosis* infection in the 3 cohorts from Vietnam, France and South Africa (333 uninfected vs 616 infected subjects) with $P < 5.0\times10^{-8}$.

Variant	EA	OR (95%CI)	*P* value	P_{het}	Chromosome	Nearest Gene
rs17155120	T	0.50 (0.45-0.55)	1.26×10^{-9}	0.31	10	*C10orf90*
rs118037357	A	0.47 (0.41-0.53)	1.89×10^{-9}	0.50	10	*C10orf90*
rs77513326	A	0.50 (0.45-0.56)	2.26×10^{-9}	0.29	10	*C10orf90*
rs79918233	A	0.51 (0.46-0.56)	5.12×10^{-9}	0.24	10	*C10orf90*
rs17155143	A	0.53 (0.48-0.58)	7.30×10^{-9}	0.10	10	*C10orf90*
rs28703703	G	0.55 (0.50-0.60)	9.18×10^{-9}	0.11	10	*C10orf90*
rs79608098	T	0.55 (0.50-0.60)	1.15×10^{-8}	0.14	10	*C10orf90*
rs75482972	A	0.55 (0.50-0.60)	1.32×10^{-8}	0.11	10	*C10orf90*
rs61750007	C	0.56 (0.51-0.61)	1.55×10^{-8}	0.12	10	*C10orf90*

CI, confidence intervals; EA, effect allele; EAF, effect allele frequency; OR, odds ratio
