# Supplementary material for: Genome-wide association study of resistance to Mycobacterium tuberculosis infection identifies a locus at 10q26.2 in three distinct populations
Source: PLoS Genet. 2021 Mar 4;17(3):e1009392. doi: 10.1371/journal.pgen.1009392 (PMC7963100; doi:10.1371/journal.pgen.1009392)
Supplement: S5 Table — (PDF) [file pgen.1009392.s021.pdf]

S5 Table. GWAS cohort in Vietnam.

	Uninfected	Infected	
	TST-/QFT-GIT-	TST+/QFT-GIT+	PTB
n	185	201	152
Age, years, mean(sd)	36(16)	32(14)	48(12)
Sex, %F	68	59	22
TST, mm, mean(sd)	1(1)	16(6)	-
QFT-GIT	negative	positive	-

PTB, pulmonary tuberculosis; QFT-GIT, QuantiFERON-TB Gold In-Tube; TST, tuberculin skin test
