# Supplementary material for: Genome-wide association study of resistance to Mycobacterium tuberculosis infection identifies a locus at 10q26.2 in three distinct populations
Source: PLoS Genet. 2021 Mar 4;17(3):e1009392. doi: 10.1371/journal.pgen.1009392 (PMC7963100; doi:10.1371/journal.pgen.1009392)
Supplement: S6 Table — (PDF) [file pgen.1009392.s022.pdf]

S6 Table. Risk factors associated with (i) double positive tuberculin skin test (5 mm cut-off) and QuantiFERON-TB Gold In-Tube results or (ii) double positive TST/QFT-GIT and pulmonary TB patients, compared to the reference group (double negative TST and QFT-GIT results) in household contacts in Vietnam.

Characteristics	TST-/QFT-GIT- n=188	TST+/QFT-GIT+ n=512	OR (95%CI)	TST+/QFT-GIT+/ PTB n=664	OR (95%CI)
Gender					
Male	61	189	ref	223	ref
Female	127	323	0.84 (0.53-1.33)	441	0.96 (0.80-1.08)
Age, years, mean(sd)	39(16)	32(14)	0.78 (0.30-1.62)	40(13)	0.93 (0.53-1.25)
BCG vaccination[a]					
No	99	260	ref	NA	NA
Yes	69	190	1.01 (0.64-1.62)	NA	NA
Number of HHC per room, mean(sd)	2.4(1.4)	2.4(1.3)	0.99 (0.81-1.20)	NA	NA
Duration of index cough before treatment, months[b]					
<2	63	193	ref	NA	NA
2-3	84	195	1.64 (0.95-2.84)	NA	NA
>3	40	120	1.07 (0.57-2.02)	NA	NA

[a]missing information for 62 individuals
[b]missing information for 5 individuals
