# Supplementary material for: Genome-wide association study of resistance to Mycobacterium tuberculosis infection identifies a locus at 10q26.2 in three distinct populations
Source: PLoS Genet. 2021 Mar 4;17(3):e1009392. doi: 10.1371/journal.pgen.1009392 (PMC7963100; doi:10.1371/journal.pgen.1009392)
Supplement: S7 Table — (PDF) [file pgen.1009392.s023.pdf]

S7 Table. Risk factors associated with both positive tuberculin skin test (5 mm cut-off) and positive interferon-γ release assay (IGRA) results compared to the reference group (negative TST and null IGRA) in household contacts in France.

**Characteristics**	**TST-/IGRA- n=33**	**TST+/IGRA+ n=147**	**OR (95%CI)**
Gender			
Male	14	72	ref
Female	19	75	0.69 (0.10-4.60)
Age, years, mean(sd)	17(21)	28(18)	**1.07 (1.01-1.15)**
Contact duration with index, log hours, mean(sd)	5(2)	5(2)	0.87 (0.46-1.51)
Index contagiousness			
High	15	79	ref
Low-intermediate	18	68	0.57 (0.02-11.11)
Tuberculosis incidence of country of birth			
High	5	51	ref
Low-intermediate	28	96	0.14 (0.05-1.37)
Complementary health insurance[a]			
No	10	45	ref
Yes	23	99	0.58 (0.01-8.33)

[a]information missing for 3 individuals
