# Supplementary material for: Genome-wide association study of resistance to Mycobacterium tuberculosis infection identifies a locus at 10q26.2 in three distinct populations
Source: PLoS Genet. 2021 Mar 4;17(3):e1009392. doi: 10.1371/journal.pgen.1009392 (PMC7963100; doi:10.1371/journal.pgen.1009392)
Supplement: S9 Table — (PDF) [file pgen.1009392.s025.pdf]

S9 Table. Risk factors associated with both positive tuberculin skin test (5 mm cut-off) and interferon-γ release assay (IGRA) results compared to the reference group (both negative TST and IGRA results) in the family-based study in South Africa.

**Characteristics**	**TST-/IGRA- n=128**	**TST+/IGRA+ n=152**	**OR (95%CI)**
Gender			
Male	60	69	ref
Female	58	83	1.22 (0.67-2.17)
Age, years, mean(sd)	10(6)	15(6)	**1.30 (1.20-1.44)**
