# Supplementary material for: Genome-wide association study of resistance to Mycobacterium tuberculosis infection identifies a locus at 10q26.2 in three distinct populations
Source: PLoS Genet. 2021 Mar 4;17(3):e1009392. doi: 10.1371/journal.pgen.1009392 (PMC7963100; doi:10.1371/journal.pgen.1009392)
Supplement: S10 Table — (PDF) [file pgen.1009392.s026.pdf]

S10 Table. GWAS cohort in South Africa.

	TST-/IGRA-	TST+/IGRA+
n	118	136
Age, years, mean(sd)	10(4)	15(6)
Sex, %F	49	56
TST, mm, mean(sd)	0(0)	17(5)
IGRA, IFN-γ, pg/mL	0	>20.9

IGRA, interferon-γ release assay; TST, tuberculin skin test
