# Supplementary material for: Genome-wide association study of resistance to Mycobacterium tuberculosis infection identifies a locus at 10q26.2 in three distinct populations
Source: PLoS Genet. 2021 Mar 4;17(3):e1009392. doi: 10.1371/journal.pgen.1009392 (PMC7963100; doi:10.1371/journal.pgen.1009392)
Supplement: Attachment — Submitted filename: Response_reviews_PGENETICS-S-20-01869.docx [file pgen.1009392.s027.docx]

Dear Editors,

Thank you very much for considering a revised version of our manuscript PGENETICS-S-20-01869 entitled “Genome-wide association study of resistance to Mycobacterium tuberculosis infection identifies a locus at 10q26.2 in three distinct populations”. As you will see from our response below, we have incorporated the changes suggested by the reviewers. Overall, this has resulted in a significantly improved version of the manuscript.

We are indebted to both reviewers who have contributed a substantial amount of their time to read, think and comment on our study, especially during these challenging times.

Below, we have reproduced the comments made by the reviewers. We have answered the questions in the order as they appear in the reviews and explain how we have changed the manuscript to take the suggestions into account.

We hope that our revised version of the manuscript is now acceptable for publication in *PLoS Genetics.*

Sincerely yours,

Dr. Aurélie Cobat, Dr. Erwin Schurr and Dr. Laurent Abel

***Reviewer #1:*** *Thank you for your interesting study and well written manuscript. Overall,
I found the report clear and comprehensive.*

We thank the reviewer for her/his positive assessment of our work.

*I have a few comments that I feel would improve the manuscript, as follows:*

*Line 161-167: It is possible that LTBI has different genetic etiology compared to PTB and yet you group the two together as infected. You do state that you additionally ran the GWAS in the subset with PTB removed and you show that the chromosome 10 SNPs remain significant. It would be interesting to see the GWAS results for the PTB removed subset, at least to see a manhattan plot comparison.*

**Reply:** As outlined by the reviewer, different genetic factors are likely to underlie resistance to TB infection and development of PTB once infected. Since PTB patients are, by definition, infected with *Mtb* we decided to include them in the group of infected individuals in order to increase the sample size and the power to identify genetic variants conferring resistance to *Mtb* infection. In order to verify that the 10q26.2 locus was a resistance locus for infection and not a locus involved in PTB, we performed the GWAS in Vietnam between double negative (n=185) and double positive (n=201) individuals only. The Manhattan plot is shown below. We did not observe any genome-wide significant association, likely due to the smaller sample size. Importantly, the 18 variants of the 10q26.2 locus were still associated with protection against infection with *P* < 5.0 × 10^-6^_,_ with similar ORs (for rs17155120, OR = 0.40, 95% CI 0.32-0.49, *P* = 2.55 × 10^-7^). In addition to the 10q26.2 locus, we could observe a cluster of SNPs on chromosome 7 (top variant rs2055017, *P* = 6.9 × 10^-8^). However, this association signal strongly dropped down when adding the PTB patients (*P* = 2.6 × 10^-5^), and there was no replication of this signal in the French and South African cohorts.

In the revised version, we added the Manhattan plot figure in the supplemental material (Figure S4), and quoted it in the manuscript line 162 (Results): “We also performed a GWAS between the 185 uninfected and the 201 infected subjects, excluding the 152 PTB patients (S4 Figure).“

*Do the French and south African cohorts include PTB subjects combined with the TST+/IGRA+ group?*

**Reply:** None of the French and South African subjects suffered of TB at the time of recruitment. In the French cohort, which is a household contact study, only the household contacts and not the index TB patients were included. In the South African study, only healthy children and young adults living in a highly endemic area for TB were included and tested for infection.
We modified the manuscript as follows:

-l 368 (Study settings and populations, South Africa): “Subjects who had clinical TB disease in the 2 years preceding the study were excluded. Thus, only healthy children and young adults from the area were included and tested for infection.”

-l 419 (Definition of the M. tuberculosis infection phenotype): “For the French study, which included only HHCs and none their index PTB index cases, contacts could have had…”

*Previous Results: What are the results for variants or regions previously identified. In the introduction you reference the studies and findings, but do not show what the association results looked like in your current study. I believe it would be informative to see what the previously identified results were in your current study.*

**Reply:** We looked for replication of the variants conferring protection from TB infection identified by genome-wide approach and fine-mapping of a linkage study (Sobota et al., Plos Genet 2017 and Igo et al., Genes Immun 2019). We selected the 10 reported variants with *P* < 5 × 10^-5^ in Sobota et al. 2017, and the chromosome 2 variant rs7568133 associated with persistent negative TST in Igo et al. 2019. None of them were replicated in the 3 cohorts (see table below). The different phenotype definition (based only on TST for the two studies) and population studied (HIV-positive in Sobota et al.) may have led to the inability to replicate the previous associations in our study.

| **Reported**  **Variant** | **Chr.** | **Vietnam** | **P_V_** | **France** | **P_Fr_** | **South Africa** | **P_SA_** |
| --- | --- | --- | --- | --- | --- | --- | --- |
| rs877356 | 5 | rs877356 | 0.68 | rs877356 | 0.86 | rs877356 | 0.80 |
| rs17062122 | 6 | rs17062122 | 0.41 | rs17062122 | 0.92 | rs17062122 | 0.10 |
| rs654718 | 11 | rs654718 | 0.60 | rs654718 | 0.92 | rs654718 | 0.53 |
| rs7944514 | 11 | rs7944514 | 0.89 | rs7944514 | 0.94 | rs7944514 | 0.96 |
| rs7837658 | 8 | rs7837658 | 0.43 | rs7837658 | 0.61 | rs7837658 | 0.34 |
| rs8142256 | 22 | rs8142256 | 0.16 | rs8142256 | 0.21 | rs8142256 | 0.51 |
| rs7808481 | 7 | rs7808481 | 0.48 | rs7808481 | 0.66 | rs7808481 | 0.03* |
| rs10998959 | 10 | rs10998959 | 0.39 | rs10998959 | 0.69 | rs10998959 | 0.27 |
| rs1880386 | 10 | rs1880386 | NA | rs1880386 | 0.57 | rs1880386 | 0.71 |
| rs11736841 | 4 | rs11736841 | 0.68 | rs11736841 | 0.14 | rs11736841 | 0.45 |
| rs7568133 | 2 | rs7568133 | 0.42 | rs7568133 | 0.68 | rs7568133 | 0.86 |
| *effect in opposite direction | | | | | | | |

*Line 399-403: Did you include PCs in your regression model? You check the association between many covariates and the phenotype, but the list of covariates does not include PCs.*

**Reply:** We performed the GWAS in a linear mixed model framework, which did not need to include PCs as covariates to account for ethnic heterogeneity (Price et al, Nat Rev Genet. 2010 or Listgarten et al, Nat Methods 2012). Population structure and familial relationships were taken into account by computing a genetic relatedness matrix from genotyped variants, which is included in the mixed model. The genomic inflation factor (λ=0.997) and the QQ plot showed that population structure and relatedness were well controlled.

*Line 399-403: You check for covariates and their association with TST+/IGRA+ vs. TST-/IGRA null. Do you check the association between the covariates and the combined phenotype (TST+/IGRA+ or PTB vs. TST-/IGRA null)?*

**Reply**: When adding PTB patients to the TST+/IGRA+ group in the Vietnamese cohort, only the effect of age and sex could be assessed since exposure covariates were not available in PTB patients. We did not observe any significant association of age and sex with the combined phenotype (S6 Table updated) leading to no further adjustment.

We modified the manuscript lines 409-418 (Definition of the M. tuberculosis infection phenotype part) as follows: “A total of 188 subjects were classified as double negative and 512 as double positive (S14 Figure), among which 185 and 201 subjects were genotyped, respectively. In order to increase the sample size, we also added 152 genotyped PTB patients, consisting of 146 index cases and 6 subjects with a history of PTB, to the infected group (S5 Table). We investigated covariates associated with our infection definition and no significant association was found (S6 Table). Therefore, we conducted an unadjusted genetic association analysis of our binary infection phenotype.”

***Reviewer #2:*** *In this paper, Quistrebert et al performed a GWAS of resistance to TB infection, using 3 distinct cohorts. First, they evaluted a sample of Vietnamese household contacts from individuals from Vietnam, followed by validation in two separate cohorts, France and South Africa. In each cohort, genetic results were adjusted for ancestral genetic architecture, age, and sex. They found a locus on 10q26.2 as strongly associated with resistance to either QFT or TST in the Vietnamese population, and validation at lower significance in the other populations. These data represent an interesting addition to the growing field of TB genetic studies, with the added novelty of evaluation of the unique "TB resister" phenotype. Therefore these results are of a good deal of interest to the field. I have some minor issues to address before publication, but generally, this is a quite well written and methodologically sound manuscript.*

We thank the reviewer for his/her positive evaluation of our work.

*Major issues; Although there was description of how resistance was defined, it would be nice to have a bit more clarity on this score, as the longer the duration of exposure to TB, the more powerful the genetic association of the phenotype becomes. I wonder if there was there a determination of an exposure score in the cohorts, as done in the Stein Ugandan cohort? Is there a way to define the amount of time under exposure to Mtb more than estimated coughing time?*

**Reply:** We agree with the reviewer that phenotype definition is a key parameter of the power of the study. For resistance to infection phenotype, it is pivotal to ensure that “resistant” individuals have been exposed to the pathogen. In our study, the resistance phenotype was assessed using both TST and IGRA results, and exposure was guaranteed by either household exposure (Vietnam and France) or high community exposure (South Africa). We did not use a score to quantify exposure as we did not have enough information for all individuals allowing to derive a robust score. However, we explored relevant covariates related to exposure when possible (e.g. BCG vaccination, duration of cough, number of individuals per household room in Vietnam ; or duration of contact with index, contagiousness of index, TB incidence in country of birth, health insurance in France ; see supplementary tables 5 and 7). Of note, households in Vietnam and France were exposed for an average of 3 months and in the study of Stein et al. 2018, a majority of conversions occurred within 3 months of exposure. As stated by the reviewer, insufficient exposure can lead to misclassifications of subjects, which can affect the power of the study but not the type I error. Hence, while it is possible that the power of our analysis may be slightly decreased, we are confident that the 10q26 locus is associated with resistance to *Mtb* infection.

*Minor issues: It appears that many of the individuals in Vietnam cohort developed active TB as compared to other HHC. How does this number compare to overall measures of progression from those individualas exposed to TB? If it is higher than expected, does that mean anything for the future analysis?*

**Reply:** We apologize if the study design of the Vietnamese cohort was not clearly defined. Index TB cases were first identified and then their household contacts were invited in the study to be further tested by both TST and IGRA. The 152 PTB cases who were added in the Vietnamese GWAS were mostly those index cases (146) and 6 individuals who had a previous history of PTB as indicated in the Methods (l 414). HHCs were not followed to assess if they developed or not active TB (for those who were infected). Thus, it was not possible to investigate risk factors for progression to TB disease and it was not the objective of our study.

We modified the manuscript l 342 (Study settings and populations, Vietnam): as follows: “HHCs did not undergo any further follow-up.”

***Reviewer #3:*** *The manuscript by Quistrebert reports an association of a region on chromosome 10q26 with resistance to tuberculosis in highly-exposed individuals, replicated in three cohorts of diverse racial/ethnic backgrounds. The study is well-designed, with rigorous analysis and quality control checks. Annotation of the association by public expression database results suggests that the variants associated with resistance might modulate a Th17 immune response.

In general, the paper is well written and the results and interpretation are convincing.*

We thank the reviewer for his/her positive evaluation of our work.

*There are a few points that the authors should address to improve the impact of the paper:
1) The definition of “highly exposed” seems to vary a bit across the three cohorts, with two appearing to depend largely on co-residence and long term exposure to a PTB case (Vietnam & France) but one not requiring such exposure (S. Africa). Could the effort to quantify or establish exposure be better described and any effort to harmonize across studies be outlined? This seems less necessary with the consistency of results, but the results in SA are the weakest, and this might be due to misclassification of individuals as “resistant, highly exposed” and attenuation of the association accordingly.*

**Reply:** This comment joins the remark of reviewer#2 and we agree that the level of exposure is a key parameter of such studies. To minimize misclassification of resistant and infected individuals, we first relied on both TST and IGRA and defined a strict phenotype which was the same across the 3 cohorts. We acknowledge that the cross-sectional testing is a limitation and that only a longitudinal follow-up with repeated assays and deep phenotyping could distinguish more precisely between individuals with persistent negative results and those who convert or revert. Exposure was not quantified but was guaranteed here by either household exposure (Vietnam and France) or high community exposure (South Africa). Indeed, in South Africa several studies showed that infection mainly occurs in the community (see Verver et al. Proportion of tuberculosis transmission that takes place in households in a high-incidence area. Lancet 2004;363:212–4 or Andrews et al. Integrating social contact and environmental data in evaluating tuberculosis transmission in a South African township. J Infect Dis2014; 210:597–603). Nevertheless, as noted by the reviewer, it is possible that some individuals were misclassified which in that case would impact on the power of the study but not on the type I error. Hence, while it is possible that the power of our analysis may be slightly decreased, we are confident that the 10q26 locus is associated with resistance to *Mtb* infection.

*2) There is no discussion of the role of HIV in these three cohorts, other to note in the introduction that seroprevalence is low overall in Vietnam. Was HIV status known in these individuals? If not, this should be discussed.*

**Reply:** We agree with the reviewer that HIV can be an important confounding factor in such studies. While screening of participants for HIV status was not possible for legal and logistical reasons, none of the participant was known to be a HIV patient. In addition, HIV seroprevalence was estimated at 0.4% in Vietnam in the general population and at 5.2% among TB cases at the time of the study (https://data.worldbank.org/indicator/SH.DYN.AIDS.ZS?locations=VN ; WHO TB report 2015). Hence, even if we cannot rule out the possibility that the HIV seroprevalence is slightly higher in household contacts of TB patients than in the general population, it should not exceed 5% in the Vietnamese cohort. The same conclusion applies in France where the HIV seroprevalence of index TB cases in our study was estimated at 7% (https://data.worldbank.org/indicator/SH.DYN.AIDS.ZS?locations=FR ; Aissa et al., Am. J. Respir. Crit. Care Med. 2007). In South Africa, children and young adults were sampled from the general population where the HIV seroprevalence was estimated at 5.2% in the overall population and less than 2% in the pediatric population at the time of the study (Cobat et al. JExpMed 2009). In addition, known HIV positive individuals were excluded from the study (Cobat et al. JExpMed 2009). Finally, immunosuppression could be indirectly assessed by the positive controls (mitogen tubes) of IGRA tests with low responses indicating an inability to respond to antigens and leading to indeterminate results. Since quality control of IGRA results were good for all individuals, HIV was unlikely to be a major confounder in our study.

We modified the manuscript as follows (Study settings and populations) :

- Vietnam, l. 336: “… 0.4% in 2015 in the general population and 5% among TB cases,[39],[41].”
- France, l. 3448-349: “… BCG vaccination rates are high[40]. HIV seroprevalence is low in France in the general population (0.3% at the time of the study [43]) and was estimated at 7% in the index cases of the study [44].“
- South Africa, l. 363-366: “…BCG vaccination at birth is routine in this area [48]. HIV seroprevalence was estimated at 5.2% in the overall population and less than 2% in the pediatric population at the time of the study,[11]. In addition, individuals who were known to be HIV positive, pregnant, or using immunomodulatory chemotherapy were excluded at the time of enrollment[11].”

*3) The use of an uncorrected p-value threshold of 0.05 for replication should be discussed and justified, since multiple variants were tested. While LD was very strong in the Vietnam sample (thus suggesting this is really one test) it is weaker between some marker pairs in the other two samples, suggesting some correction might be necessary. To be sure, Bonferroni correction is too stringent, but an estimate of the number of independent tests is possible, and perhaps advisable here.*

**Reply:** We agree with the reviewer that applying an uncorrected 0.05 p-value threshold when multiple loci are tested for replication is not advisable. Here we considered that only one locus was tested given the high LD in Vietnam among the genome-wide significant SNPs. However, as pointed by the reviewer the LD is slightly weaker in France and South Africa compared to Vietnam where two LD blocks are inferred by Haploview, as shown in supplementary figure 5. Assuming 2 independent blocks were tested, the corrected p-value threshold is 0.025 which doesn’t change the main findings (p-values for the genotyped variant rs17155120 are 0.015 and 0.017 in France and South Africa).

We modified the manuscript as follows:

- Methods, l 461-469: “Replication of genome-wide associated variants (*P* < 5 × 10^-8^) in the primary cohort from Vietnam was assessed in the two cohorts from France and South Africa. The observed LD in France and South Africa was slightly weaker as compared with Vietnam, and two LD blocks were inferred by Haploview (S5 Figure). We therefore considered variants at a nominal one sided *P* value < 0.025 and with a consistent direction of the effect size as replicated.”
- Results, l 190 to 199: “In the French cohort, 17 variants of the cluster could be genotyped or imputed (including 11 genome-wide significant variants), and 3 were replicated at the P < 0.025 level.” […] “In the South African population, 12 variants of the locus were genotyped or successfully imputed (including 10 genome-wide significant variants) and 3 of them showed evidence for replication.”

*4) While the authors indicate that data are publicly available, the doi provided lists summary statistics for the 3 datasets. PLoS Genetics data availability policy states that large-scale data (such as GWAS) should be made available in a public repository upon publication. The authors should discuss whether this is possible, and if not, explain why only summary statistics will be provided.*

**Reply:** We apologize for the incorrect statement on data availability. The different studies started more than 10 years ago and the consent forms did not include at that time an information about the possibility to sharing of individual level genetic data outside the study investigators. Sharing of data is only permitted in the context of studies of TB pathogenesis which precludes the depositing in a pubic data base. Therefore, only summary statistics can be provided. However, data are available from the corresponding author upon request for studies dealing with TB pathogenesis.

We updated the manuscript in Availability of data and material l 496 : “All summary statistics are available in the manuscript or at https://doi.org/10.5281/zenodo.3942125. Because of the IRB restriction on the data from Vietnam, France and South Africa, individual level data are only available in the context of a specific collaboration on tuberculosis research upon request from the authors.“
